# Time-efficient and computer-guided sprint interval exercise training for improving health in the workplace: a randomised mixed-methods feasibility study in office-based employees

**DOI:** 10.1186/s12889-020-8444-z

**Published:** 2020-03-12

**Authors:** Richard S. Metcalfe, Hady Atef, Kelly Mackintosh, Melitta McNarry, Gemma Ryde, Denise M. Hill, Niels B. J. Vollaard

**Affiliations:** 1 Applied Sports Science, Technology, Exercise and Medicine Research Centre (A-STEM), Swansea University, Swansea, SA1 8EN UK; 2 Department of Physical Therapy for Internal Medicine, Faculty of Physical Therapy, Cairo University, Giza, Egypt; 3 Faculty of Health Sciences and Sport, University of Stirling, Stirling, FK9 4LA UK

**Keywords:** Exercise, High-intensity interval training, Workplace health, Effectiveness, Feasibility, Acceptability, Cardiorespiratory fitness

## Abstract

**Background:**

The efficacy of high-intensity interval training (HIT) as a time-efficient exercise strategy for beneficially modifying risk factors for cardiovascular disease has repeatedly been demonstrated in controlled laboratory settings. However, the effectiveness of HIT in an unsupervised workplace setting has not been investigated. The objective of this study was to use mixed methods to investigate the feasibility, acceptability and effectiveness of a short-duration, high-intensity exercise intervention (REHIT) when applied unsupervised in a workplace setting.

**Methods:**

Twenty-five office-workers (mean ± SD age: 47 ± 9 y, BMI: 27.5 ± 4.4 kg·m^− 2^, V̇O_2_max: 28 ± 7 mL·kg^− 1^·min^− 1^) completed a 6-week REHIT intervention unsupervised in their workplace (*n* = 13, 6 men), or acted as a no-intervention control (*n* = 12, 6 men). The intervention consisted of 2 sessions/week of low-intensity (~ 25 W) cycling interspersed with 2 ‘all-out’ sprints, increasing in duration from 10 to 20 s per sprint over the 6 weeks (total time-commitment: 8:40 min per session). V̇O_2_max was assessed pre- and post-training, whilst questionnaire-based measures of exercise enjoyment, self-efficacy, and acceptability were completed post-training. Eight participants also completed post-intervention semi-structured interviews.

**Results:**

V̇O_2_max significantly improved in the exercise group (2.25 ± 0.75 L·min^− 1^ vs. 2.42 ± 0.82 L·min^− 1^; + 7.4%) compared to the control group (2.22 ± 0.72 L·min^− 1^ vs. 2.17 ± 0.74 L·min^− 1^; − 2.3%; time*intervention interaction effect: *p* < 0.01). Participants considered the REHIT intervention acceptable and enjoyable (PACES: 89 ± 17 out of 119) and were confident in their ability to continue to perform REHIT (7.8 ± 1.2 out of 9). Qualitative data revealed that REHIT offered a time-efficient opportunity to exercise, that was perceived as achievable, and which encouraged highly valued post-exercise outcomes (e.g. progress towards health/fitness benefits).

**Conclusions:**

REHIT could be implemented as a feasible, effective and acceptable exercise intervention in a workplace setting, with a total time-commitment of < 20 min/week. Consideration of certain psycho-social factors and behaviour-change techniques may ensure adherence to the REHIT programme in the long term.

**Trial registration:**

The study was registered on ClinicalTrials.gov on 07/05/2019 (registration: NCT03941145).

## Introduction

Increasing the proportion of the population taking part in regular exercise has been described as ‘the best buy for public health’ [1]. In support of this view, there is a wealth of descriptive literature demonstrating that various exercise interventions can, on average, acutely and/or chronically modify key cardiometabolic risk biomarkers in a direction that would be likely to reduce the risk of chronic disease [2–4]. Concordantly, long-term intervention studies have provided causal evidence that regular exercise either alone, or in combination with other lifestyle interventions (e.g. dietary modifications), reduces the risk of developing type 2 diabetes by ~ 50% [5, 6], whilst large associational studies consistently report a dose-dependent reduction in cardiovascular and all-cause mortality in people self-reporting, or objectively measured, as being more physically active [7]. The strong and causal relationship between higher levels of cardiorespiratory fitness (V̇O_2_max) and lower rates of cardiovascular and all-cause mortality further emphasises the importance of regular exercise [8], as this is the only potential public health intervention capable of enhancing V̇O_2_max.

The first position stand and guidelines for aerobic exercise were published in 1975, have been updated frequently since, and currently recommend at least 150 or 75 min/week of moderate- or vigorous-intensity aerobic activity, or a combination of the two [9–11]. However, the proportion of adults meeting these guidelines is typically poor, with studies reporting adherence rates of between 5 and 47% when measured objectively [12–14]. Furthermore, over the last 3–4 decades, there has been a striking deterioration in cardiorespiratory fitness within the adult working population [15, 16].

Whilst the reasons for low exercise uptake and adherence are complex, one commonly reported barrier is a ‘lack of time’ [17]. This can be addressed by developing more time-efficient exercise interventions. It is therefore unsurprising that the last decade has seen a substantial number of research studies focusing on higher-intensity / higher-effort exercise paradigms aiming to achieve health benefits with shorter exercise and shorter total time commitments [18]. The majority of this work has concentrated on cardiorespiratory exercise modalities in which the exercise bouts are applied in short intervals of (sub) maximal (‘high-intensity interval training’ [HIIT]) or supramaximal (‘sprint interval training’ [SIT]) intensity exercise bouts, interspersed with periods of low-intensity or resting recovery. There is now a convincing body of evidence demonstrating that, at least in the short-term, both HIIT and SIT are associated with very similar, and in some cases superior, cardiometabolic health benefits compared with currently recommended moderate-intensity continuous training (MICT [19–21];). However, multiple sprint repetitions combined with the need for recovery intervals mean that many HIIT and SIT protocols are not as time-efficient as often claimed, with most taking ~ 22–40 min per training session [19]. The reduction in positive affect during HIIT/SIT is also directly dependent on the number and duration of high-intensity intervals [22], with one of the main critiques of HIIT/SIT being that negative affective responses may deter unfit or sedentary populations from adhering to it in the long-term [23–25].

In response to this critique and based on hypotheses on the underlying mechanisms responsible for adaptations to SIT, we have previously conducted a series of studies which collectively demonstrate that both the number of sprint repetitions and the sprint duration can be reduced without attenuating the associated health benefits [19]. The resulting low volume SIT protocol (termed ‘reduced-exertion high-intensity interval training’; REHIT) consists of two 20-s ‘all-out’ sprints within a 10-min otherwise low-intensity exercise session, including warm-up, recovery between sprints and cool-down [26]. We have demonstrated improvements in key cardiometabolic biomarkers with REHIT, including V̇O_2_max [26–29], insulin sensitivity and glycaemic control [26, 30] and blood pressure [31]. Importantly, REHIT is genuinely time-efficient (2 x ~ 10-min per week) and the low number of sprint repetitions required means it is less likely that participants will experience negative affective responses during exercise [32]. As a result, participants may be more likely to adhere to the exercise programme in the longer-term. However, whilst there is accumulating evidence for efficacy of REHIT to improve cardiometabolic health in supervised laboratory settings, it now needs to be investigated whether this can be replicated in an unsupervised and real-world situation (i.e. ‘effectiveness’).

The workplace has been specifically identified as an ideal location to target health promotion interventions aimed at preventing chronic disease because of the potential to access a large proportion of the adult population [33]. In theory, providing convenient and flexible access to facilities for exercise in the workplace could overcome many important barriers to exercise, including poor facilities, lack of transportation, personal costs, and bad weather [17, 34]. In reality, workplace exercise interventions have generally, although not universally, been shown to result in small improvements in employee physical activity levels [35–37], whilst interventions promoting physical activity specifically of moderate-to-vigorous intensity appear to positively impact cardiorespiratory fitness [38]. However, the majority of workplace exercise interventions involve significant employee time commitment [38], and perceived lack of time availability due to busy work schedules has been identified as a predictor of poor long-term engagement in workplace exercise initiatives [39]. With the potential positive effects of REHIT in lab-based studies and its short duration, it is essential that its effectiveness is tested in this context. Therefore, the aim of this feasibility study was to perform a randomised controlled trial and utilise mixed methods to investigate the acceptability and effectiveness of REHIT when applied in an unsupervised workplace setting.

## Methods

### Participants

**Fig. 1:**
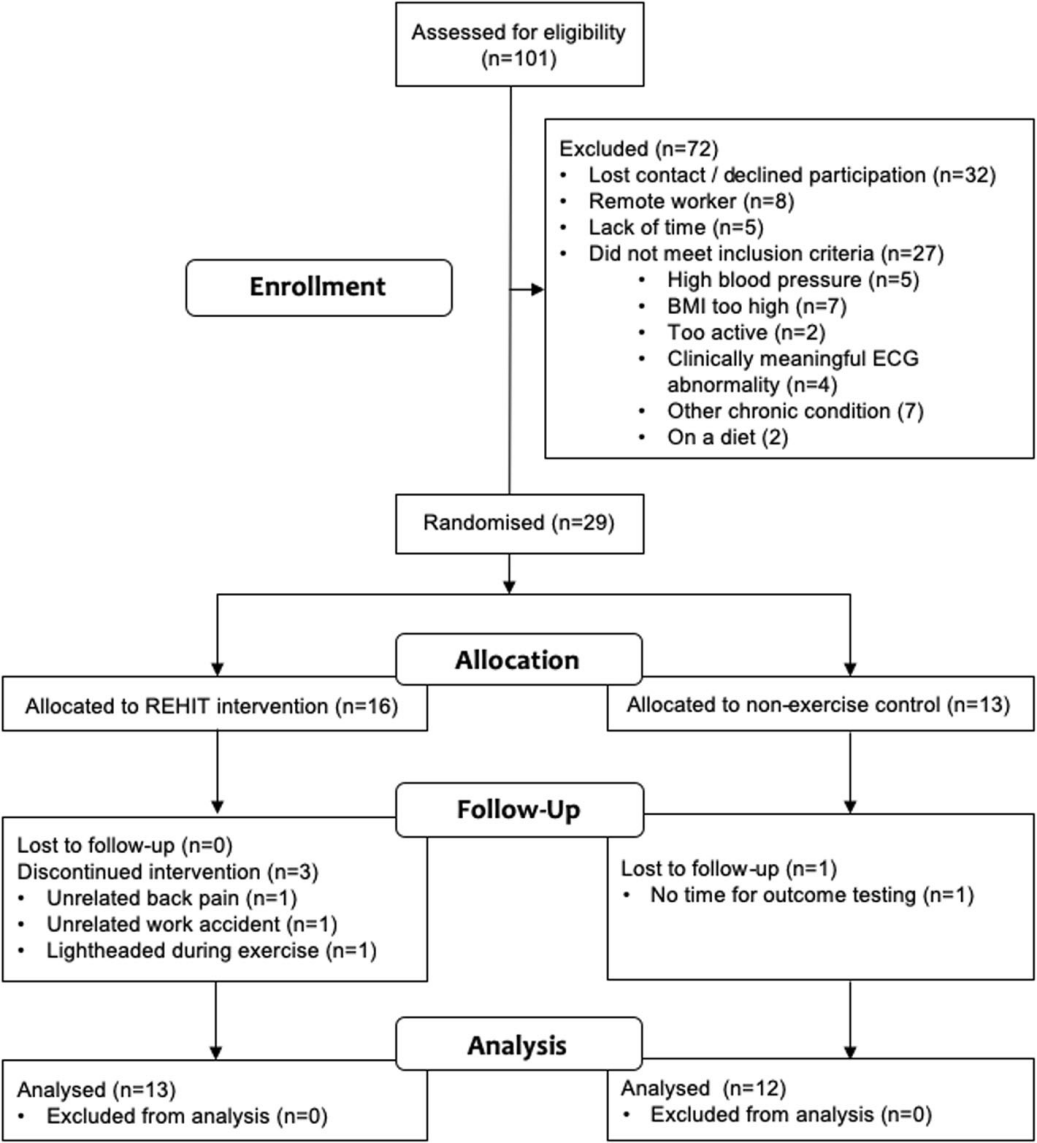
Flow of participants through the study

Employees aged > 18 years and < 60 years who were employed in an office-based job consisting of mainly sedentary tasks at the two participating workplaces (Local Government Authorities in Central Scotland and South Wales, UK) were recruited to take part in this randomised controlled trial (Fig. 1). We aimed to recruit between 24 and 50 participants, as for feasibility trials it has been recommended that a minimum of 12 participants per arm is appropriate [40]. Exclusion criteria were a history of type 2 diabetes, insulin therapy, cardiovascular disease, cerebrovascular disease, use of β-blockers, the answer ‘yes’ to any questions on a standard physical activity readiness questionnaire (PARQ), classification as highly physically active according to the scoring criteria of the International Physical Activity Questionnaire (IPAQ), a clinically significant resting ECG abnormality, uncontrolled hypertension (systolic blood pressure > 140 mmHg and/or diastolic blood pressure > 90 mmHg after at least a 5-min seated rest), and BMI > 35 kg·m^− 2^. The study was advertised via workplace email channels and on their local intranet between April and June 2019. Employees interested in participating in the study were initially screened for the inclusion/exclusion criteria via a telephone interview, and subsequently invited for a more thorough screening session prior to baseline tests. Twenty-nine male (*n* = 12) and female (*n* = 17) volunteered to take part. Following baseline measures, participants were randomised to an exercise group (performing 6 weeks of REHIT in their own workplace; *n* = 16; *n* = 10 at Swansea and *n* = 6 at Stirling, respectively), or a no-exercise control group (*n* = 13; *n* = 7 at Swansea and n = 6 at Stirling, respectively). Randomisation was performed using the sealed envelope method. Four participants dropped out of the study (exercise group: *n* = 3 from Swansea; provided reasons: unrelated back-pain, unrelated work accident, and light-headedness following exercise; control group: n = 1 from Swansea; provided reason: lack of time to attend testing sessions). Participant characteristics of the final sample (*n* = 25; 12 male) included in the analysis are provided in Table 1. Within the final sample, 21 participants scored low on the IPAQ and 4 were classified as moderately active. There were no significant differences in age, body mass, BMI, V̇O_2_max or physical activity (IPAQ) between the control group and the exercise group at baseline (Table 1). Ethical approval was provided by the relevant local University ethics committees (University of Stirling: NICR 18/19–036; Swansea University: 2019–022) and the experiment was conducted in accordance with the Helsinki declaration. All participants provided written informed consent prior to participation. The study was registered on ClinicalTrials.gov (registration: NCT03941145).

**Table 1:** Participant characteristics

	Control (***n*** = 12)	Exercise (***n*** = 13)
**Sex (male / female)**	6 / 6	6 / 7
**Age (y)**	48 ± 8	46 ± 9
**Body Mass (kg)**	79.8 ± 13.7	80.0 ± 13.8
**BMI (kg·m**^**− 2**^**)**	27.9 ± 4.4	27.2 ± 4.5
**Baseline V̇O**_**2**_**max (mL·kg**^**− 1**^**·min**^**− 1**^**)**	28 ± 7	28 ± 7
**Physical activity level (MET-min·week**^**− 1**^**)**	339 ± 355	542 ± 520

*Values shown are means ± SD. Physical activity level was determined using the IPAQ*

### Study design

Participants were invited for testing sessions at baseline and after the 6-week intervention period, both of which took place at the exercise physiology laboratories at either Swansea University or the University of Stirling. Participants were randomised following the baseline testing session. Participants randomised to the exercise group completed post-training assessments 3-days following the final exercise session, whilst participants randomised to the no-intervention control group attended a post-intervention assessment approximately 6 weeks following baseline testing. The primary outcome, and our main measure of effectiveness of the intervention, was the change in maximal aerobic capacity (V̇O_2_max). Quantitative psychological questionnaires for exercise motivations, perceived stress and health-related quality of life were secondary outcome measures of effectiveness. Participants in the exercise group also completed questionnaires related to exercise enjoyment and intervention acceptability (secondary outcomes). Semi-structured interviews (secondary outcome) to further explore the acceptability of the intervention were conducted in a sub sample of employees allocated to the exercise group and took place during the week following the post-training testing session.

### Physiological outcomes

*Maximal aerobic capacity (V̇O*_*2*_*max):* The primary outcome measure was the change in V̇O_2_max from baseline to post-intervention, as determined using an incremental cycling test to volitional exhaustion (Excalibur Sport, Lode, Groningen, the Netherlands), with breath-by-breath measurement of oxygen uptake using a calibrated online gas analyser (Swansea: Jaegar Vyntus, Vyaire Medical; Stirling: Cortex Metalyzer, Cortex). Participants were requested to refrain from performing strenuous exercise and consuming caffeine and alcohol the day before testing, and to drink half a litre of water the morning of the testing day. After a two-min warm-up at 20 W the intensity was increased by 1 W every 3 s until volitional exhaustion (failure to maintain rpm > 50 despite verbal encouragement). V̇O_2_max was determined as the highest value of a 15-breath rolling average and a maximal effort was accepted if ≥2 of the following criteria were met: i) volitional exhaustion, ii) a plateau in V̇O_2_ despite increasing intensity, iii) Respiratory exchange ratio > 1.15, and iv) maximal heart rate within 10 beats of the age-predicted maximum (i.e., 220-age). This was the case for all participants.

### Quantitative psychological outcomes

#### Exercise task self-efficacy

Participants’ confidence in their ability to continue the REHIT intervention for a further 6-week period was assessed using a 1-item measure, adapted from McAuley et al. [41], which asked ‘*How confident are you that you can perform two bouts of exercise that are just like the ones you have completed, each week for the next six weeks?*’. Responses were scored on a scale of 1 (Not at all confident) to 9 (Extremely confident).

#### Enjoyment

A modified version of the Physical Activity Enjoyment Scale (PACES [42]) was used to examine enjoyment of the REHIT intervention in participants allocated to the exercise group. Similar to Jung et al. [43], the PACES scale was modified by removing 1 irrelevant item (*‘I was very / not at all absorbed in the activity*’). Instead of the original PACES instructions (‘*please rate how you feel at the moment about the physical activity you have been doing*’), participants were asked to ‘*think about the exercise routine you did for the study and rate your enjoyment of it’*. The 17 items were scored on a 7-point bipolar scale, resulting in an overall enjoyment score between 17 (not enjoyable), 68 (neutral) and 119 (enjoyable). Again, similar to Jung et al. [43], two additional questions were asked: ‘*how much did you enjoy the exercise sessions you completed for this study?*’, and ‘*how much do you think you would enjoy performing exercise sessions just like the ones you completed, two times per week for the next six weeks?*’. These additional questions were scored on a 9-point Likert scale ranging from 1 (I did/would not enjoy it at all) to 9 (I enjoyed/would enjoy it very very much) and analysed individually.

#### Acceptability

Acceptability of the REHIT training programme was assessed using the 11-item questionnaire by Boereboom et al. [44], scored on a 5-point Likert scale from 1 (strongly disagree) to 5 (strongly agree). Items were analysed individually.

#### Perceived stress

Changes in perceived stress were assessed using the Perceived Stress Scale (PSS [45]) in all participants. The PSS includes 10 items rated on a 5-point scale ranging from 0 (never) to 4 (very often), resulting in a total perceived stress score out of 40.

#### Motivation for physical activity and exercise

The RM 4-FM: Motivation for Physical Activity Questionnaire [46] was used to examine changes in the extent of extrinsic and intrinsic motivation to engage in physical activity or exercise in all participants. Participants indicated their reasons for performing physical activity (16 items) or exercise (12 items) on a 7-item scale ranging from 1 (not at all true), to 7 (very true), providing two overall Relative Autonomy Index scores indicating the relative impact of intrinsic and extrinsic factors in the motivation to be active, with more positive values reflecting higher intrinsic motivation.

#### Health-related quality of life (HRQoL

Changes in HRQoL were examined using the 36-item short-form questionnaire (SF-36) in all participants. The SF-36 questionnaire evaluates self-reported health status, function, with lower scores indicating a higher degree of disability [47].

### Qualitative interviews

A subsample of eight participants (1 male) completed individual interviews via Skype or phone post-intervention (20–30 min in duration). One interviewee had withdrawn from the study (end of week 3), and the remainder had completed the 6-week programme. Interviews were utilized to generate an understanding of the participants’ experience of the REHIT programme, which may have influenced their engagement and adherence. Hence, the interview schedule (Additional File 1) aimed to explore: i) overall experience of the REHIT exercise programme; ii) motives for initially taking part in the programme; iii) reasons for remaining / or for not remaining committed to the programme; and iv) intention to maintain exercise behaviour beyond the intervention.

### Exercise intervention

Participants allocated to the exercise group were asked to perform two REHIT exercise sessions per week for six weeks. Each session involved a low-intensity warm-up (2 min at ~ 25 W), two 20-s ‘all-out’ cycle sprints against a resistance equivalent to 5% of body mass, and 3 min of low-intensity recovery (~ 25 W) following each sprint. Sprint duration increased from 10 s during the first 3 sessions, to 15 s during sessions 4–6, and 20 s during the remaining 6 sessions. Maximum total exercise time was 8 min and 40 s. The exercise intervention was delivered on a commercially available cycle ergometer (CAR.O.L™, Integrated Health Partners Ltd., London, UK), which can be delivered unsupervised. The bike was placed within the workplace environment, and a privacy screen was used to prevent exercise being in full view of colleagues. A computer screen on the handlebars provided guidance on what to do throughout each training session, with an option for headphones to receive the information in audio-format. Participants were required to log in on the bike’s screen with a personal code, which enabled adherence to be monitored remotely. Although no specific behaviour change techniques were incorporated into the intervention, the bike’s computer programme does provide participants with real-time power output and heart rate data throughout each session, and feedback on (potential) improvements in peak power output during the session is provided on session completion. Peak power output during the sprints was recorded for each session for the purposes of analysis. Participants in the no-intervention control group were asked to continue their normal lifestyle for the duration of the study and were offered the opportunity to perform the REHIT intervention following completion of post-intervention testing.

### Statistical analysis

Data are presented as mean ± SD. Statistical analysis was performed using SPSS Statistics 23 (INM, Chicago, USA). Normality of the data was confirmed using the Shapiro-Wilk test. Differences in baseline characteristics were compared using an independent sample t-test. Differences between the groups for changes in V̇O_2_max (the primary outcome measure), changes in body mass, perceived stress, relative autonomy index for physical activity and for exercise, and general health were analysed using two-way mixed-model analysis of variance [intervention*time]. Differences in peak power output averaged over the first and last training weeks were analysed using a paired sample t-test. Significance was accepted at *p* < 0.05.

The qualitative data were analysed via directed content analysis in which a deductive and inductive approach was adopted to describe the phenomenon for the purpose of extending existing knowledge and theory [48]. Therefore, key themes, concepts and variables were identified within the interviewees’ data, for the purpose of describing and explaining their experience of the 6-week REHIT programme. More specifically, each transcript was line-by-line coded, with codes placed within the relevant overarching category (i.e. overall experience of the REHIT exercise programme, motives for initially taking part in the programme, reasons for remaining / or for not remaining committed to the programme, and intention to maintain exercise behaviour beyond the intervention). Similar / opposing codes were then organised into themes, which collectively provided a descriptive account of the interviewees’ experience (i.e. time-appeal, positive or neutral perception of exercise bouts, highly negative perceptions of exercise bouts, and adherence maintained).

## Results

### Feasibility: adherence and intervention Fidelity

On average, participants in the exercise group completed > 90% of the prescribed REHIT sessions. Six participants completed all 12 sessions (100% adherence), four missed one session (92% adherence), two missed two sessions (83% adherence) and one missed four sessions (67% adherence). All participants were retained in the analysis. The mean peak power achieved during the all-out sprints was 532 ± 79 W, corresponding to 2.8 ± 0.5 times the power output achieved during the V̇O_2_max test. Peak power output during the REHIT sessions increased from an average of 508 ± 179 W during week 1 to an average of 545 ± 188 W during week 6 (*p* < 0.05).

### Effectiveness: physiological and psychological effects of REHIT

**Fig. 2:**
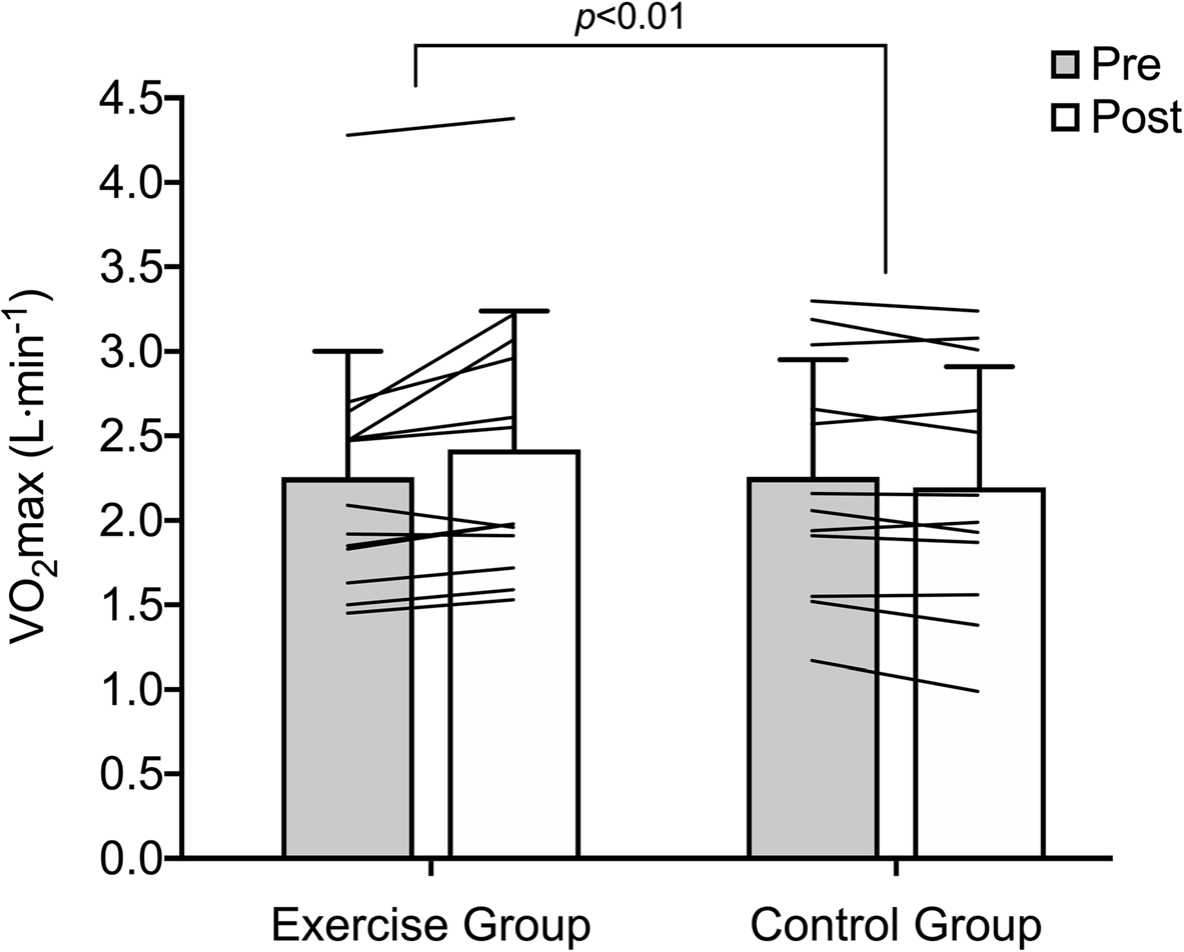
Changes in maximal aerobic capacity over the intervention period. Grey bars: baseline, white bars: post-intervention. Overlaid lines represent individual responses

There was a significant increase in absolute V̇O_2_max in the exercise group (2.25 ± 0.75 L·min^− 1^ vs. 2.42 ± 0.82 L·min^− 1^; + 7.4%) compared to the control group (2.26 ± 0.70 L·min^− 1^ vs. 2.20 ± 0.71 L·min^− 1^; − 2.3%; time*intervention interaction effect: *p* < 0.01; Cohen’s *d* effect size: 1.4; Fig. 2). No significant changes in body mass were observed (control group: 79.8 ± 13.7 vs. 79.7 ± 14.1 kg; exercise group: 80.0 ± 13.8 vs. 80.7 ± 13.7 kg). The relative autonomy index for physical activity did not change, irrespective of group, but there was an increase in the relative autonomy index for exercise in the REHIT group compared with the control group over the 6-week intervention (*p* < 0.05 for the group*time interaction effect; Table 2). Perceived general health increased after 6 weeks (*p* < 0.05 for main effect of time) with no differences between the REHIT group and the control group (Table 2). Perceived stress remained unchanged in both groups after six weeks (Table 2).

**Table 2:** Changes in psychological outcomes over the intervention period

	Control group (***n*** = 12)		Exercise group (***n*** = 13)	
	Baseline	Post	Baseline	Post
Perceived Stress Scale (PSS)	19.4 ± 3.1	20.0 ± 3.2	19.7 ± 3.3	18.8 ± 3.2
Relative autonomy index for physical activity (RM 4-FM)	11.3 ± 2.0	9.5 ± 2.5	9.3 ± 3.1	10.0 ± 2.9
Relative autonomy index for exercise (RM 4-FM)	4.6 ± 3.0	3.6 ± 2.0^b^	3.3 ± 4.1	4.6 ± 5.3^b^
Health-Related Quality of Life: general health (SF-36)	60 ± 14	67 ± 11^a^	53 ± 17	61 ± 16^a^

*a: p < 0.01 for the main effect of time*

b: p < 0.05 for the time*intervention interaction effect

### Acceptability: quantitative outcomes

On average, participants agreed with statements that the REHIT intervention was enjoyable, that they were confident they could continue REHIT for another 6 weeks (and continue to enjoy it), and that they would recommend REHIT to others (Table 3). They disagreed with statements that REHIT had interfered with other aspects of their life due to the time commitment and physical strain involved (Table 3).

**Table 3:** Task self-efficacy, enjoyment, and acceptability of the REHIT intervention

Task self-efficacy ^**a**^	7.8 ± 1.2
Enjoyment	
*Enjoyment of REHIT exercise sessions*^***a***^	7.5 ± 1.5
*Expected future enjoyment of REHIT exercise sessions*^***a***^	7.2 ± 1.9
*Modified PACES*^***b***^	89.1 ± 16.6
Acceptability ^**c**^	
*“Doing REHIT has been enjoyable”*	4.4 ± 0.8
*“I would recommend REHIT to others”*	4.6 ± 0.5
*“REHIT has been physically more demanding than I expected”*	3.3 ± 1.3
*“I would be happy to continue to do REHIT”*	4.5 ± 0.8
*“Doing REHIT has interfered with other aspects of my life due to the time commitment”*	1.5 ± 0.7
*“Doing REHIT has interfered with other aspects of my life due to the travelling involved”*	1.8 ± 1.4
*“Doing REHIT has interfered with other aspects of my life due to the physical strain”*	1.6 ± 1.0
*“I believe REHIT has improved my fitness”*	3.7 ± 0.6
*“I am pleased to have done something to improve my fitness”*	4.5 ± 0.9
*“I would have preferred to exercise in a group setting”*	2.5 ± 1.0
*“I would have preferred to exercise at home”*	2.5 ± 1.1

a: scored on a scale of 1 (not at all confident/enjoyable) to 9 (extremely confident/enjoyable)

b: overall enjoyment score from 17 (not enjoyable at all) to 119 (enjoyable)

c: scored on 5-point Likert scale from 1 (strongly disagree) to 3 (neither agree nor disagree) to 5 (strongly agree)

### Acceptability: qualitative experiences

A summary of the key themes arising from the participant interviews are shown in Table 4. All interviewees indicated they were initially motivated to take part in the REHIT exercise workplace-based programme because of the perceived opportunity to gain health / fitness benefits within the time-limited constraints of their working and personal lives. One participant summarised how REHIT could be integrated into a busy life, in order to achieve their health and fitness benefits:

“*I don’t have the time to do any structured exercise … I commute to work each day … and when I get home, I cook tea, take the kids to activities, and then it’s 9 o’clock and I haven’t done anything but sit in the car … I need to stay fit, well, and alive! I know I don’t do enough exercise, but I don’t have enough time to do it … But there is no excuse to get out of work for half an hour and do this [REHIT]”* [P5].

**Table 4:** Themes arising from the qualitative interviews

Stage of protocol	Theme	Sub-theme
REHIT adopted / initiated	Time-appeal	Health and fitness benefits
		Limited time commitments required
		High self-efficacy due to brief exercise bouts
		Convenient location
Early stages of the exercise programme (≤15 s)	Positive perception of exercise bouts	Exercise bouts perceived as achievable
		Positive psycho-social responses post-exercise
Later stages 20 s)	Highly negative perceptions of exercise bouts	Exercise bouts perceived as intensely demanding
		Increasing reluctance to engage with exercise
		Positive feedback regarding progress towards health / fitness goals
	Adherence maintained	The use of cognitive and behavioural strategies
		Positive feedback
		Positive psycho-social responses post-exercise
		Habit
		Intention to commit long-term

Therefore, the required time commitments of REHIT, alongside the convenience of locating the exercise bike within the workplace, was suggested to be of critical importance for exercise adoption and potential longer-term maintenance:

“*All of the barriers, that normally are there … you know, mentally and practically … were taken away. So, this [REHIT] was something I could do in work time, and still enjoy the [health / fitness] benefits [P8]”.*

Moreover, while the interviewees anticipated the intensity of the REHIT exercise bouts would be demanding, they all indicated high levels of exercise self-efficacy prior to the intervention, which was then maintained through the early stages of the REHIT protocol (duration ≤15 s). That is, they felt physically capable of completing the protocol and the shorter (≤15 s) exercise bouts. As explained by one participant:

“*If you can’t do something for 10 minutes twice a week and put effort in … then there is something wrong*” [P7]. While another stated, “… *going as fast as you can for the 10 seconds, well I can do that!! … that is manageable … I just saw it as two minutes*...*”.* [P4].

The interviewees recalled that during the shorter bouts of exercise (≤15 s) of the REHIT protocol, they all experienced positive affect (e.g. *enjoyment, feeling comfortable, excited,* and *interested*). However, once the duration of the exercise bout extended to 20 s, such affectual responses became increasingly negative for seven of the eight interviewed participants (e.g. *discomfort, anxious,* and *unease*). They perceived the exercise had become difficult / strenuous, which, in turn, appeared to lower their exercise self-efficacy. This finding was highlighted by one of the participants:

*“I began to feel that there was just no way I was going to be able to do the 20 seconds flat flat out... I was just like, I just can’t, I just can’t keep this up!... It was a huge step up, and … it made me kind of wobble and doubt myself”* [P1].

Nevertheless, seven interviewees completed the 6-week programme, with only one withdrawing (out of the four that withdrew from the overall study). They reported adopting several self-generated strategies during the exercise bouts to temper any negative affect associated with the exercise and enhance motivation. This included distraction (i.e. shutting eyes, listening to music, counting down), motivational self-talk, and the use of social support from an exercise ‘buddy’:

“*I did it with a colleague, so we did it together. That helped buoy you up when needed, and keep you going” [P5].*

In addition, receiving positive feedback regarding their progress from the computer including how their peak power had increased was also reported as a critical motivating factor. One of the interviewees explained this point further:

“… *each week you could see your peak was up 2%. So in my mind, I’m getting fitter, and I will see if I can then go up again the following week … the feedback kept me going and going*” [P7].

Of note, it was also inferred by a number of the interviewees [3], that as the study progressed to its latter stages, the exercise routine became more habitual. Therefore, any reluctance (and associated inertia) to attend the exercise session, became easier to overcome.

Throughout the REHIT exercise intervention, all interviewees suggested they had experienced a considerable sense of achievement, and a feeling of being energised. In some cases, this state may have held the potential to increase work productivity (if exercise was completed in the morning/lunch time). As an example, one participant recalled that after completing the REHIT session, she felt:

*… knackered, but then absolutely buzzing … and those endorphins pretty much lasted the whole day. You just feel more alive, more energized … you’ve got more spring in the step … you just feel more awake … I tend to get a bit of an afternoon slump, and on those days, I noticed I didn’t have it so much. So yeah … I must have been more productive [P1]*

Finally, six of the eight interviewees (including the participant who withdrew from the study), indicated they would like to continue with the REHIT exercise programme post-intervention. They indicated that any associated negative affect/experience of engaging with the intense exercise programme could be counteracted by the convenience of REHIT, achieving health/fitness gains, and achieving the positive psycho-social outcomes (e.g. sense of mastery, socialising and energizing). Moreover, it was also stressed by a number of the interviewees, that due to their busy lifestyle, there was no alternative exercise mode available to them:

“*I know I need to exercise, and this [REHIT] is the only chance that I’m going to have … I like paddle-boarding and whatever, but I can’t fit it in. At least this would give me something” [P5].*

For the smaller number of interviewees (*n* = 2) who suggested they would not continue with REHIT, one indicated that they preferred exercising outdoors, while the other felt unable/unwilling to engage further with REHIT, as the “*small*” physiological improvements he gained, did not justify the effort:

“*I got to be honest, I was really struggling during the exercise... I felt like it was killing me … then, because there weren’t brilliant outcomes, I thought … it was all for nothing*” [P6].

## Discussion

The potential use of HIIT and SIT in the primary prevention of chronic disease is controversial, with several recent commentaries and CrossTalk discussions criticising these types of interventions as likely suffering from ‘*limited reach, effectiveness, and adoption, and poor implementation and maintenance’* [25, 49, 50]. However, to date, few protocols have been examined in real-world settings, partly due to protocols being too long or too strenuous, and partly because suitable specialist equipment has not been available [19]. The present randomised controlled trial demonstrates for the first time that an unsupervised computer-guided REHIT intervention, involving minimal sprint durations and repetitions, can feasibly be delivered in the workplace, and can improve maximal aerobic capacity (V̇O_2_max), a key health marker, with a minimal weekly time commitment of less than 20 min (2 × 8 min 40 s per week). Importantly, the training sessions were well adhered to and deemed acceptable for participants to complete around their daily work schedules. These findings are novel and of potential significance because they demonstrate, for the first time, the translational potential of time-efficient, feasible, sprint interval exercise as a workplace exercise intervention for improving health.

Poor levels of cardiorespiratory fitness – measured directly as maximal oxygen uptake (V̇O_2_max) – are one of the strongest prognostic biomarkers for cardiovascular, cancer and all-cause mortality [8]. In a series of laboratory-based studies involving a pooled cohort of 104 sedentary participants (62 men, 42 women), our research has demonstrated that REHIT improves V̇O_2_max by 0.25 ± 0.24 L·min^− 1^ (+ 9.5%) over a 6-week intervention period [26, 27, 29]. In these studies, the exercise sessions have been supervised by an exercise physiologist with participants being provided with guidance and encouragement throughout. A legitimately raised question has been whether the fidelity and efficacy of REHIT can be replicated when performed unsupervised in a ‘real-world’ setting. During the all-out sprints in this study, participants achieved peak power outputs approximately 2.8-fold higher than they achieved during the V̇O_2_max test and this is similar to that observed in participants of similar age and body composition in a supervised laboratory-controlled environment [30]. This provides at least indirect evidence that the fidelity of the ‘all-out’ sprints involved in REHIT (i.e. the ability of participants to achieve true all-out effort) can be maintained when delivered unsupervised. The effect of REHIT on V̇O_2_max in the present study (intervention – control) was + 9.7% (+ 2.8 ml·kg^− 1^·min^− 1^) and hence these data also provide the first evidence to suggest that the efficacy of REHIT to improve V̇O_2_max following supervised and unsupervised (i.e. effectiveness) REHIT can be similar. This magnitude of V̇O_2_max increase is comparable to that reported in previous workplace exercise training studies [38], and following a HIIT intervention performed as part of a group class in a supervised gym environment [51], but greater than a recent HIIT intervention performed at home [52]. This could be of clinical relevance based on epidemiological evidence demonstrating that a ~ 3–4 ml·kg·min^− 1^ (~ 1-MET) improvement in V̇O_2_max corresponds with a 19% reduction in cardiovascular mortality and a 15% reduction in all-cause mortality over 8 years [53]. Importantly, continued improvements in V̇O_2_max would be anticipated to accrue with REHIT beyond the 6-week training programme applied in this study [54]. Thus, if adhered to, the longer-term relevance for chronic disease risk may be of even greater significance.

The combined quantitative and qualitative psychological components of the study provide a unique insight into participants’ perceptions of REHIT (i.e. acceptability) in previously inactive, unfit individuals and, by extension, provide several lessons for future research. Interestingly, alongside the pursuit of health and fitness benefits [55], participants suggested that a major reason for initiating the exercise regime (study participation) was the limited time commitment required, combined with the convenient location of the bike, meaning that their perceived self-efficacy to complete the exercise intervention was high. Although commonly reported in the literature [17], some have questioned whether ‘lack of time’ is truly a barrier to exercise participation, instead suggesting that time for exercise is just not prioritised over other activities. Whilst this may be the case, our qualitative data indicate that, at least for a certain proportion of inactive working individuals, providing a time-efficient and convenient exercise option may facilitate exercise initiation. The post-intervention interviews suggested that participants also had high levels of self-efficacy to complete REHIT prior to starting the intervention. This was mainly suggested to be based on how short the exercise was anticipated to be, despite acknowledgement of the high intensities required. This suggests the time-efficiency of REHIT is not only important for convenience, but may also heighten self-efficacy, which is known to be important for exercise adoption and, indeed, long-term adherence [56].

The early stages of the REHIT programme with shorter sprints (10–15 s) were perceived to be associated with neutral or positive affect, whereas following the progression to 20-s sprints (week 3), the majority of the participants reported a progressively more negative affective experience. However, this was not of a comparable magnitude to the very strong negative affective responses associated with other, longer HIIT/SIT protocols [23]. It is also important to note that this did not impact adherence for the majority, demonstrating that, at least in the short-term, other factors (e.g. positive feedback, use of dissociation/motivational strategies such as music and self-talk, or the support of an exercise ‘buddy’) can maintain exercise behaviour when the during-exercise affect is negative. Indeed, both the qualitative and quantitative data identified that participants generally held the intention to continue the exercise programme in the longer term, given the opportunity. Nevertheless, this did lead to negative perceptions about the end stages of the REHIT intervention, which would be anticipated to lead to exercise drop-out over the longer-term [57]. In light of this, a potential suggestion for longer-term workplace studies would be to prescribe a more gradual increase in sprint duration (i.e. more sessions at 10 s and 15 s) or perhaps even limit sprint durations to 15 s until participants feel confident and motivated to progress to 20 s. This may [29], or may not [58], attenuate the improvements in V̇O_2_max in the early stages of training, but if it limits exposure to negative affect and facilitates longer-term adherence then this would not necessarily be a concern. It would also allow time for training-induced improvements in affective responses to accrue [32]. Consistent with previous research [29], post-exercise perceived affect was highly positive, and included (primarily) a sense of achievement, feeling energised, and an increased (subjective) work productivity. Post-exercise affect is considered to influence exercise adherence less than during-exercise affect [59, 60], but these responses were still considered of value by the participants.

It is accepted that further longitudinal research is required to establish whether the generally positive perceptions of REHIT would lead to exercise adherence in the longer-term. Moreover, it is also acknowledged that to promote exercise adherence, the ‘ideal’ scenario would be to facilitate exercise behaviour that is overtly pleasant/enjoyable, rather than using cognitive, behavioural and social strategies to alleviate a more negative affective experience, in order to make the exercise ‘tolerable’ [57]. However, it is also necessary to recognise that for those participants interviewed, alternative exercise and physical activity options were not viable - regardless of how pleasant and enjoyable – due to the time-constraints of their busy lives. Thus, with lack of time, whether real or perceived, remaining the most reported key barrier to exercise, the REHIT protocol appears to offer a feasible exercise *option* that can contribute to population health and fitness. The key recommendation for further (longitudinal) work is to better integrate behaviour change techniques and theory into intervention development. Most of the techniques mentioned in the current study were generated by participants or were an unintended consequence of the computer programme used. Future studies should look to enhance these aspects of the intervention and build in more robust psychological techniques in order to create a more sustainable workplace exercise strategy. This should aim to ensure that the benefits outweigh the costs through, for example: i) goal setting and generation of regular positive feedback regarding progress towards their valued exercise goal, ii) the exercise being as convenient and brief as possible, but which still brings about the positive post-exercise physiological and psychological responses and outcomes, iii) the negative affect associated with the exercise bout being alleviated as much as possible (disassociation strategies), and iv) self-efficacy being maintained as high as possible throughout the exercise programme, through positive feedback and manageable progression.

There are a number of limitations to the current study which should be considered. Firstly, the small sample size and relatively short intervention period of 6 weeks means that inferences on the long-term adherence and effectiveness of REHIT cannot be made. Furthermore, this study only included V̇O_2_max as a biomarker of cardiometabolic health and it will be important for future studies to establish the effectiveness of REHIT for improving other health markers (e.g. body composition, blood pressure, glycaemic control). It is also worth noting that, although baseline VO_2_max and BMI were similar between the groups, the intervention group reported (non-significantly) higher mean baseline physical activity levels and it is possible this might have influenced their perceptions of the intervention. Finally, it is important to acknowledge that both the intervention and control participants were from the same workplace and there is a potential risk of contamination with this approach.

## Conclusions

In conclusion, we show here for the first time that REHIT may be a feasible, effective and acceptable workplace exercise intervention for improving cardiovascular health and fitness in office-based workers. This study provides the groundwork to inform the development of a large multi-centre randomised controlled trial with long-term follow up.

## Supplementary information

**Additional file 1.** Overview of Interview Schedule (with prompts)

## Data Availability

The datasets used and/or analysed during the current study are available from the corresponding author on reasonable request.

## Abbreviations

BMI: Body Mass Index
HIT: High-Intensity Interval Training
HRQoL: Health Related Quality of Life
IPAQ: International Physical Activity Questionnaire
MICT: moderate intensity continuous exercise
PACES: Physical activity enjoyment scale
PAR-Q: Physical activity readiness questionnaire
PSS: Perceived stress scale
REHIT: Reduced exertion high-intensity interval training
SIT: Sprint interval training
VO_2_max: Maximal oxygen uptake / cardiorespiratory fitness

## References

[1] MacAuley D, Bauman A, Frémont P (2015). Exercise: not a miracle cure, just good medicine. BMJ.

[2] Neufer PD, Bamman MM, Muoio DM, Bouchard C, Cooper DM, Goodpaster BH (2015). Understanding the cellular and molecular mechanisms of physical activity-induced health benefits. Cell Metab.

[3] Pedersen BK, Saltin B (2015). Exercise as medicine - evidence for prescribing exercise as therapy in 26 different chronic diseases. Scand J Med Sci Sports.

[4] Hawley JA, Hargreaves M, Joyner MJ, Zierath JR (2014). Integrative biology of exercise. Cell.

[5] Knowler WC, Barrett-Connor E, Fowler SE, Hamman RF, Lachin JM, Walker EA (2002). Reduction in the incidence of type 2 diabetes with lifestyle intervention or metformin. N Engl J Med.

[6] Pan XR, Li GW, Hu YH, Wang JX, Yang WY, An ZX (1997). Effects of diet and exercise in preventing NIDDM in people with impaired glucose tolerance. The Da Qing IGT and diabetes study. Diabetes Care.

[7] Ekelund U, Tarp J, Steene-Johannessen J, Hansen BH, Jefferis B, Fagerland MW (2019). Dose-response associations between accelerometry measured physical activity and sedentary time and all cause mortality: systematic review and harmonised meta-analysis. BMJ.

[8] Ross R, Blair SN, Arena R, Church TS, Després JP, Franklin BA (2016). Importance of assessing cardiorespiratory fitness in clinical practice: a case for fitness as a clinical vital sign: a scientific statement from the American Heart Association. Circulation.

[9] Garber CE, Blissmer B, Deschenes MR, Franklin BA, Lamonte MJ, Lee IM (2011). American College of Sports Medicine position stand. Quantity and quality of exercise for developing and maintaining cardiorespiratory, musculoskeletal, and neuromotor fitness in apparently healthy adults: guidance for prescribing exercise. Med Sci Sports Exerc.

[10] Blair SN, LaMonte MJ, Nichaman MZ (2004). The evolution of physical activity recommendations: how much is enough?. Am J Clin Nutr.

[11] UK Chief Medical Officers. UK Chief Medical Officers’ Physical Activity Guidelines. London: Department of Health and Social Care; 2019.

[12] Colley RC, Garriguet D, Janssen I, Craig CL, Clarke J, Tremblay MS (2011). Physical activity of Canadian adults: accelerometer results from the 2007 to 2009 Canadian health measures survey. Health Rep.

[13] Troiano RP, Berrigan D, Dodd KW, Mâsse LC, Tilert T, McDowell M (2008). Physical activity in the United States measured by accelerometer. Med Sci Sports Exerc.

[14] Marsaux CFM, Celis-Morales C, Hoonhout J, Claassen A, Goris A, Forster H (2016). Objectively measured physical activity in European adults: cross-sectional findings from the Food4Me study. PLoS One.

[15] Lamoureux NR, Fitzgerald JS, Norton KI, Sabato T, Tremblay MS, Tomkinson GR (2019). Temporal trends in the cardiorespiratory fitness of 2,525,827 adults between 1967 and 2016: a systematic review. Sports Med.

[16] Ekblom-Bak E, Ekblom Ö, Andersson G, Wallin P, Söderling J, Hemmingsson E (2019). Decline in cardiorespiratory fitness in the Swedish working force between 1995 and 2017. Scand J Med Sci Sports.

[17] Korkiakangas EE, Alahuhta MA, Laitinen JH (2009). Barriers to regular exercise among adults at high risk or diagnosed with type 2 diabetes: a systematic review. Health Promot Int.

[18] Gibala MJ, Little JP, Macdonald MJ, Hawley JA (2012). Physiological adaptations to low-volume, high-intensity interval training in health and disease. J Physiol.

[19] Vollaard NB, Metcalfe RS (2017). Research into the health benefits of Sprint interval training should focus on protocols with fewer and shorter sprints. Sports Med.

[20] Nightingale TE, Metcalfe RS, Vollaard NB, Bilzon JL (2017). Exercise guidelines to promote Cardiometabolic health in spinal cord injured humans: time to raise the intensity?. Arch Phys Med Rehabil.

[21] Gillen JB, Gibala MJ (2014). Is high-intensity interval training a time-efficient exercise strategy to improve health and fitness?. Appl Physiol Nutr Metab.

[22] Frazão DT, de Farias Junior LF, Dantas TC, Krinski K, Elsangedy HM, Prestes J (2016). Feeling of pleasure to high-intensity interval exercise is dependent of the number of work bouts and physical activity status. PLoS One.

[23] Dekker E, Ekkekakis P (2017). More efficient, perhaps, but at what price? Pleasure and enjoyment responses to high-intensity interval exercise in low-active women with obesity. Psychol Sport Exerc.

[24] Brand R, Ekkekakis P (2018). Affective-reflective theory of physical inactivity and exercise: foundations and preliminary evidence. Ger J Exerc Sport Res.

[25] Hardcastle SJ, Ray H, Beale L, Hagger MS (2014). Why sprint interval training is inappropriate for a largely sedentary population. Front Psychol.

[26] Metcalfe RS, Babraj JA, Fawkner SG, Vollaard NB (2012). Towards the minimal amount of exercise for improving metabolic health: beneficial effects of reduced-exertion high-intensity interval training. Eur J Appl Physiol.

[27] Metcalfe RS, Tardif N, Thompson D, Vollaard NB (2016). Changes in aerobic capacity and glycaemic control in response to reduced-exertion high-intensity interval training (REHIT) are not different between sedentary men and women. Appl Physiol Nutr Metab.

[28] Vollaard NBJ, Metcalfe RS, Williams S (2017). Effect of number of sprints in an SIT session on change in V˙O2max: a meta-analysis. Med Sci Sports Exerc.

[29] Nalçakan GR, Songsorn P, Fitzpatrick BL, Yüzbasioglu Y, Brick NE, Metcalfe RS (2018). Decreasing sprint duration from 20 to 10 s during reduced-exertion high-intensity interval training (REHIT) attenuates the increase in maximal aerobic capacity but has no effect on affective and perceptual responses. Appl Physiol Nutr Metab.

[30] Metcalfe RS, Fitzpatrick B, Fitzpatrick S, McDermott G, Brick N, McClean C (2018). Extremely short duration interval exercise improves 24-h glycaemia in men with type 2 diabetes. Eur J Appl Physiol.

[31] Ruffino JS, Songsorn P, Haggett M, Edmonds D, Robinson AM, Thompson D (2017). A comparison of the health benefits of reduced-exertion high-intensity interval training (REHIT) and moderate-intensity walking in type 2 diabetes patients. Appl Physiol Nutr Metab.

[32] Songsorn P, Brick N, Fitzpatrick B, Fitzpatrick S, McDermott G, McClean C (2019). Affective and perceptual responses during reduced-exertion high-intensity interval training (REHIT). Int J Sport Exerc Psychol.

[33] Dishman RK, Oldenburg B, O’Neal H, Shephard RJ (1998). Worksite physical activity interventions. Am J Prev Med.

[34] Trost SG, Owen N, Bauman AE, Sallis JF, Brown W (2002). Correlates of adults’ participation in physical activity: review and update. Med Sci Sports Exerc.

[35] Proper KI, Koning M, van der Beek AJ, Hildebrandt VH, Bosscher RJ, van Mechelen W (2003). The effectiveness of worksite physical activity programs on physical activity, Physical Fitness, and Health. Clin J Sport Med.

[36] Abraham C, Graham-Rowe E (2009). Are worksite interventions effective in increasing physical activity? A systematic review and meta-analysis. Health Psychol Rev.

[37] Chen TTL, Magnussen CG, To QG, To KG (2013). Workplace physical activity interventions: a systematic review. Am J Health Promot.

[38] Burn NL, Weston M, Maguire N, Atkinson G, Weston KL (2019). Effects of workplace-based physical activity interventions on cardiorespiratory fitness: a systematic review and meta-analysis of controlled trials. Sports Med.

[39] Muir SD, Silva SSM, Woldegiorgis MA, Rider H, Meyer D, Jayawardana MW (2019). Predictors of success of workplace physical activity interventions: a systematic review. J Phys Act Health.

[40] Julious SA (2005). Sample size of 12 per group rule of thumb for a pilot study. Pharm Stat.

[41] McAuley E (1993). Self-efficacy and the maintenance of exercise participation in older adults. J Behav Med.

[42] Kendzierski D, DeCarlo KJ (1991). Physical activity enjoyment scale: two validation studies. J Sport Exerc Psychol.

[43] Jung ME, Bourne JE, Little JP (2014). Where does HIT fit? An examination of the affective response to high-intensity intervals in comparison to continuous moderate- and continuous vigorous-intensity exercise in the exercise intensity-affect continuum. PLoS One.

[44] Boereboom CL, Phillips BE, Williams JP, Lund JN (2016). A 31-day time to surgery compliant exercise training programme improves aerobic health in the elderly. Tech Coloproctol.

[45] Cohen S, Kamarck T, Mermelstein R (1983). A global measure of perceived stress. J Health Soc Behav.

[46] Deci E, Ryan R. The “what” and “why” of goal pursuits: Human needs and the self-determination of behavior. Psych Inq. 2000;11(4):227–68.

[47] Ware JE, Sherbourne CD (1992). The MOS 36-item short-form health survey (SF-36). I. Conceptual framework and item selection. Med Care.

[48] Hsieh H-F, Shannon SE (2005). Three approaches to qualitative content analysis. Qual Health Res.

[49] Holloway TM, Spriet LL (2015). CrossTalk opposing view: high intensity interval training does not have a role in risk reduction or treatment of disease: CrossTalk. J Physiol.

[50] Biddle SJ, Batterham AM (2015). High-intensity interval exercise training for public health: a big HIT or shall we HIT it on the head?. Int J Behav Nutr Phys Act.

[51] Shepherd SO, Wilson OJ, Taylor AS, Thøgersen-Ntoumani C, Adlan AM, Wagenmakers AJ (2015). Low-volume high-intensity interval training in a gym setting improves cardio-metabolic and psychological health. PLoS One.

[52] Roy M, Williams SM, Brown RC, Meredith-Jones KA, Osborne H, Jospe M (2018). High-intensity interval training in the real world: outcomes from a 12-month intervention in overweight adults. Med Sci Sports Exerc.

[53] Lee DC, Sui X, Artero EG, Lee IM, Church TS, McAuley PA (2011). Long-term effects of changes in cardiorespiratory fitness and body mass index on all-cause and cardiovascular disease mortality in men: the aerobics center longitudinal study. Circulation.

[54] Gillen JB, Martin BJ, MacInnis MJ, Skelly LE, Tarnopolsky MA, Gibala MJ (2016). Twelve weeks of Sprint interval training improves indices of Cardiometabolic health similar to traditional endurance training despite a five-fold lower exercise volume and time commitment. PLoS One.

[55] Ingledew DK, Markland D (2008). The role of motives in exercise participation. Psychol Health.

[56] Biddle SJH, Nigg CR (2000). Theories of exercise behavior. Int J Sport Psychol.

[57] Ekkekakis P, Dafermos M. Exercise Is a Many-Splendored Thing, but for Some It Does Not Feel So Splendid: Staging a Resurgence of Hedonistic Ideas in the Quest to Understand Exercise Behavior. Oxf Handb Exerc Psychol. 2012 May 11 [cited 2019 Oct 15]; Available from: https://www.oxfordhandbooks.com/view/10.1093/oxfordhb/9780195394313.001.0001/oxfordhb-9780195394313-e-16.

[58] Zelt JG, Hankinson PB, Foster WS, Williams CB, Reynolds J, Garneys E (2014). Reducing the volume of sprint interval training does not diminish maximal and submaximal performance gains in healthy men. Eur J Appl Physiol.

[59] Zenko Z, Ekkekakis P, Ariely D (2016). Can you have your vigorous exercise and enjoy it too? Ramping intensity down increases Postexercise, remembered, and forecasted pleasure. J Sport Exerc Psychol.

[60] Neef NA, Shade D, Miller MS (1994). Assessing influential dimensions of reinforcers on choice in students with serious emotional disturbance. J Appl Behav Anal.

