# Supplementary material for: Time-efficient and computer-guided sprint interval exercise training for improving health in the workplace: a randomised mixed-methods feasibility study in office-based employees
Source: BMC Public Health. 2020 Mar 12;20:313. doi: 10.1186/s12889-020-8444-z (PMC7068982; doi:10.1186/s12889-020-8444-z)
Supplement: Supplementary file 1 — Additional file 1. Overview of Interview Schedule (with prompts) [file 12889_2020_8444_MOESM1_ESM.docx]

**Overview of Interview Schedule (with prompts)**

- Previous history of exercise / PA / Sport
  - Motives for (non)participation
    - [Prompts: why]
- Before the programme
  - Perception of exercise
  - Expectations of protocol
  - Concerns / aims / hopes
    - [Prompts: why]
- During the exercise
  - Perceptions
  - Positives
  - Challenges
  - Strategies to adhere / overcome
  - Motives to adhere
  - Factors affecting engagement
  - Any changes in the above
    - [Prompts: how / why]
- Post-protocol
  - Overall reflections of the exercise programme?
  - Enjoyment
  - Easier / harder than thought
  - Expectations
  - Outcomes
  - Positives / challenges / expectations / aims met
  - Future exercise intentions
  - Changes in exercise motives
    - [Prompts: why (physical competence / efficacy etc) / how?]
- Any impact on work and productivity (positive/negative) and why?
- Suggestions for changes / improvements to the intervention – and why / how?
- Potential long-term adherence to this exercise intervention if equipment was available – why / why not?
